# From reference genomes to population genomics: comparing three reference-aligned reduced-representation sequencing pipelines in two wildlife species

**DOI:** 10.1186/s12864-019-5806-y

**Published:** 2019-06-03

**Authors:** Belinda Wright, Katherine A. Farquharson, Elspeth A. McLennan, Katherine Belov, Carolyn J. Hogg, Catherine E. Grueber

**Affiliations:** 1 Faculty of Science, The University of Sydney, School of Life and Environmental Sciences, Sydney, Australia; 2 San Diego Zoo Global, San Diego, USA

**Keywords:** Population genomics, DArTseq, Reference genome, Tasmanian devil, Pink-footed goose, Population differentiation, Stacks, SAMtools, GATK

## Abstract

**Background:**

Recent advances in genomics have greatly increased research opportunities for non-model species. For wildlife, a growing availability of reference genomes means that population genetics is no longer restricted to a small set of anonymous loci. When used in conjunction with a reference genome, reduced-representation sequencing (RRS) provides a cost-effective method for obtaining reliable diversity information for population genetics. Many software tools have been developed to process RRS data, though few studies of non-model species incorporate genome alignment in calling loci. A commonly-used RRS analysis pipeline, Stacks, has this capacity and so it is timely to compare its utility with existing software originally designed for alignment and analysis of whole genome sequencing data. Here we examine population genetic inferences from two species for which reference-aligned reduced-representation data have been collected. Our two study species are a threatened Australian marsupial (Tasmanian devil *Sarcophilus harrisii*; declining population) and an Arctic-circle migrant bird (pink-footed goose *Anser brachyrhynchus*; expanding population). Analyses of these data are compared using Stacks versus two widely-used genomics packages, SAMtools and GATK. We also introduce a custom R script to improve the reliability of single nucleotide polymorphism (SNP) calls in all pipelines and conduct population genetic inferences for non-model species with reference genomes.

**Results:**

Although we identified orders of magnitude fewer SNPs in our devil dataset than for goose, we found remarkable symmetry between the two species in our assessment of software performance. For both datasets, all three methods were able to delineate population structure, even with varying numbers of loci. For both species, population structure inferences were influenced by the percent of missing data.

**Conclusions:**

For studies of non-model species with a reference genome, we recommend combining Stacks output with further filtering (as included in our R pipeline) for population genetic studies, paying particular attention to potential impact of missing data thresholds. We recognise SAMtools as a viable alternative for researchers more familiar with this software. We caution against the use of GATK in studies with limited computational resources or time.

**Electronic supplementary material:**

The online version of this article (10.1186/s12864-019-5806-y) contains supplementary material, which is available to authorized users.

## Background

Decreasing sequencing costs and increasing availability of genomic resources mean that population genetic studies are more often utilising genomic data. Whereas in the past tens of microsatellites may have been used to infer population structure and answer fundamental and applied questions, now thousands of single nucleotide polymorphisms (SNPs) can be generated and aligned to reference genomes [1, 2]. Reduced-representation sequencing (RRS), also referred to as genotyping-by-sequencing (GBS), or restriction-site associated DNA sequencing (RADseq, also ddRAD), is an approach to generate genome-wide high-throughput sequencing data [3, 4]. This is achieved by reducing the genomic data to be sequenced using restriction enzyme digestion and next-generation sequencing (NGS) of the resultant fragments [4]. While RRS provides a cost-effective method of sequencing a large number of genome-wide loci across many individuals, coupling this approach with an assembled reference genome improves the reliability of genotype calls [5] and subsequently improves any downstream inferences [6].

One of the initial benefits of RRS approaches was the lack of a need for a reference genome [4]. However, now that the costs of generating reference genomes are declining, genetics researchers may take a top-down approach, whereby the genome sequencing project is undertaken first to provide the scaffold for later population genetic studies using RRS (e.g. [7–9]). In this context, biologists who start with a reference assembly may develop familiarity with, and in-house pipelines for, bioinformatic software designed for whole genome sequencing (WGS), such as SAMtools [10] and the Genome Analysis Toolkit (GATK) [11]. While these software can be used for analysing RRS data, specialist tools such as Stacks [12] are purpose built for RRS, and designed for use with or without a reference genome [12–14]. In practice, the algorithms underlying software tools for analysing WGS versus RRS data can differ considerably, which in turn may influence conclusions drawn. For example, calibration of GATK SNP calling parameters is highly dependent on known variant datasets [15], making parameterisation problematic for non-model species.

Many studies have found major differences in resultant datasets produced using various WGS [16, 17] or RRS [5] software tools, but none have specifically compared the analysis of reference-aligned RRS data in Stacks versus two widely used genome software packages, SAMtools and GATK. This knowledge gap has been noted by the software developers themselves [14] and so our study serves to fill this gap. Furthermore, comparisons between analysis tools have focused largely on computational efficiency and the total number of SNPs obtained [5, 18] and few have examined the critical problem of whether biological interpretations of real data are affected by alternate pipelines [6]. This application is important, because fundamental genomic differences between threatened and non-threatened species (such as variation in levels of diversity, inbreeding and linkage disequilibrium) have the potential to impact our analytical choices, inference, and the transferability of population genetic findings [19]. As concerns over the current biodiversity crisis deepen, there has been a call for the greater use of genetic and genomic data in the management of species both in captivity and the wild [20, 21].

In this study, we employed three widely-used programs, Stacks, SAMtools and GATK, to call variants from reference-aligned RRS data collected from two species with very different demographic histories, and determine how differences between these analysis pipelines impact population interpretations across contexts. Our first study species is a threatened Australian marsupial, the Tasmanian devil *Sarcophilus harrisii* (hereafter “devil”). The devil has exhibited a severe population crash due to the emergence of a contagious cancer, devil facial tumour disease (DFTD) in the 1990s [22, 23]. To aid conservation of the species, the devil genome was sequenced in 2012 [24]. We generate RRS data from devil samples and anticipate moderate population structure between wild devils of western and eastern Tasmania origin, based on previous analyses using microsatellites [25, 26] and genomics [27, 28]. Our second study species is the pink-footed goose *Anser brachyrhynchus* (hereafter “goose”), which breeds in the Arctic and overwinters in Northern Europe and has a reference genome available [29]. For the goose, we reanalyse a subset of the data made available by Pujolar et al. [8]. Their study used population genetic analyses to examine connectivity between two putatively separate populations and infer the effects of climate and human activities on demography of this migrant species. The purpose of our analysis here is not to specifically recapitulate the population genetic investigations for these two species. Rather, we aim to discover how inferences in two very different species, both with known population structure, are impacted by variation in analysis tools.

## Results

### Within-population diversity

**Fig. 1:**
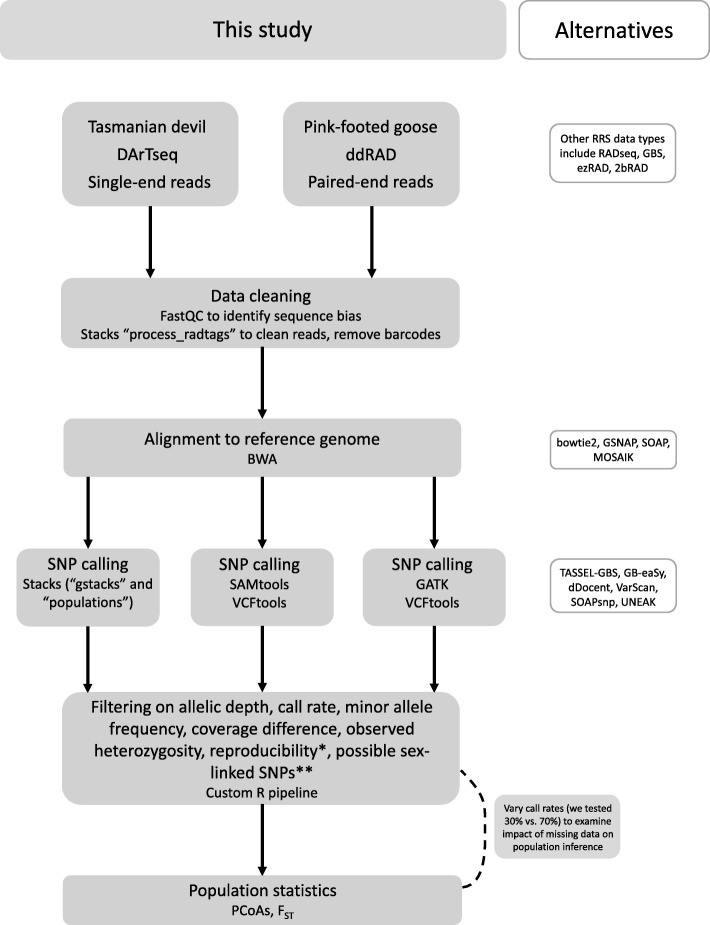
Overview of methods used in this study to process reduced representation sequencing data with reference genomes, with some alternatives to software used indicated where appropriate. * Reproducibility filtering only possible if replicates or technical replicates are performed. ** Possible sex-linked SNP filter requires knowledge of sex of samples and is based on XX/XY system, but could be reversed for ZZ/ZW systems

We applied our three analysis pipelines (Stacks, SAMtools, GATK; all further processed with the custom R script; Fig. 1) to a total of 131 devil samples and 40 goose samples. Our main results focus on two major study populations of each species, which were expected to show genetic differentiation (devil [25–28]; goose [8]). The devil dataset also contains a third population of captive individuals which are mixed provenance between east and west [30]. We used this latter population to test how well each analysis pipeline discriminates among populations with mixed lineages.

Mean sequencing coverage was similar for both species, although more variable for geese. Mean cover for devils was 12.8x (S.D. = 3.5, range = 7.8–32.0). For geese mean cover was 13.3x (S.D = 6.7, range = 1.4–26.4). Unsurprisingly, considering the demographic histories of the two species, the number of SNPs returned for each differed substantially (Table 1), although we acknowledge that the laboratory methods for the two datasets were also different (see [8]; Additional file 1: Supplementary Methods). After all filtering steps (including a 70% call rate), the GATK pipeline obtained the highest number of SNPs for both species: 1464 for devil and 277,362 for goose. Stacks returned a similar number of SNPs as GATK for devils, while Stacks and SAMtools approaches returned a substantially smaller number of SNPs than GATK for goose (Table 1); we note we used the same stringency cut-offs for all three data processing pipelines, as far as the user-definable parameters of each software permitted (Fig. 1).

**Table 1:** Summary statistics for the resultant SNP loci datasets of three pipelines, filtered at a 70% call rate (see Additional file 1: Table S1 for data filtered on 30% call rate), for Tasmanian devil (*N* = 131) and pink-footed goose (*N* = 40), including total number of loci (total loci), average number of loci sequenced across individuals (mean loci), amount of missing data (%), calculated error rates (%), mean observed heterozygosity across loci (H_O_), mean expected heterozygosity across loci (H_E_), and average multilocus heterozygosity of individuals (MLH)

Dataset	Pipeline	CPU hours^a^	Total loci	Mean loci (min; max)	% missing	Error rate (%)^b^	H_O_ (± SD)	H_E_ (± SD)	MLH (± SD)
Devil	Stacks	16	1359	1177.3 (500; 1326)	13.4	2.9	0.207 (0.149)	0.248 (0.163)	0.205 (0.043)
	SAMtools	55	251	205.8 (96; 236)	18.0	6.6	0.308 (0.160)	0.327 (0.115)	0.298 (0.092)
	GATK	325	1464	1297.2 (604; 1442)	11.4	5.3	0.185 (0.139)	0.256 (0.161)	0.184 (0.040)
Goose	Stacks	11	52,053	44,914.4 (954; 50,517)	13.7	NA	0.132 (0.127)	0.156 (0.136)	0.127 (0.026)
	SAMtools	14	26,437	22,035.0 (732; 23,732)	16.7	NA	0.256 (0.160)	0.307 (0.142)	0.563 (0.158)
	GATK	65	277,362	245,412.2 (6787; 270,0084)	11.5	NA	0.137 (0.121)	0.187 (0.149)	0.132 (0.034)

^a^CPU hours represent total computational time for each pipeline excluding alignment and the further filtering in R. Note that while some steps can be parallelised for quicker computation, not all steps allow for this

^b^Error rates could not be calculated for the pink-footed goose dataset as no replicates were included in the current analysis. Error rate is calculated after filtering on SNPs with > 85% reproducibility, so is lower than initial error rates

For both species, mean multilocus heterozygosity estimates obtained using Stacks and GATK were noticeably lower than for SAMtools (Table 1). Genotype ratios (ratios of genotypes called as either of the two homozygotes or as heterozygotes) were similar between species but varied across pipelines (Additional file 1: Figure S1). SAMtools was more likely to call heterozygous genotypes than either Stacks or GATK, explaining the higher heterozygosity estimates for SAMtools (Table 1). Stacks was more likely to call the most common homozygote (Additional file 1: Figure S1).

By aligning our datasets to reference genomes we were able to unambiguously identify each SNP based on its genomic position and determine the degree of consistency among the three analysis methods. For devil, across all three pipelines, a total of 2060 unique SNPs were identified; 155 (7.5%) of these were identified by all three methods (Additional file 1: Figure S2a). For goose, this pattern was similar: 78,235 unique SNPs were identified, of which 3283 (4.2%) were common to all three methods (Additional file 1: Figure S2b). Concordance rates between genotype calls across pipelines, calculated according to shared loci, were high (Table 2). Concordance rates were slightly higher for devils (for which SNPs were filtered on their reproducibility; see below) than goose (where no replicates were performed so the error rate could not be reduced). Concordance was also higher between both Stacks and GATK and Stacks and SAMtools than for GATK and SAMtools for both species (Table 2). Comparing the genotypes that differed between samples across the different pipelines, Stacks was more likely to call a genotype heterozygous that was called homozygous in either SAMtools or GATK. There were very few homozygous to alternate homozygous discordant genotype calls between all pipelines (Table 2).

**Table 2:** Genotypic differences between loci common to the 3 pipelines for devils (155 loci) and geese (3283 loci). Concordance rates (identical genotype calls between samples) between pipelines are in parentheses. Discordant genotype calls are presented as the percent of total genotypes

	Stacks:SAMtools	Stacks:GATK	GATK:SAMtools
Devil	(97.77)	(98.15)	(98.92)
Homozygous → Homozygous	0.00005	0.00010	0.00005
Homozygous → Heterozygous	0.00039	0.00227	0.00197
Heterozygous → Homozygous	0.01719	0.01369	0.00670
Goose	(97.06)	(97.64)	(97.85)
Homozygous → Homozygous	0.00019	0.00018	0.00043
Homozygous → Heterozygous	0.00395	0.00628	0.00394
Heterozygous → Homozygous	0.01920	0.01355	0.01327

Homozygous → Homozygous refers to those loci where an AA is called a TT in the other pipeline for example. Homozygous → Heterozygous are any genotype calls that are homozygous in the first pipeline but called heterozygous in the other for that sample at the same locus. Heterozygous → Homozygous are those calls that are heterozygous in one pipeline but called homozygous in the other for that sample at the same locus

For devil only, a subset of 35 individuals were sequenced twice, allowing us to compare the reproducibility of genotype calls from our three pipelines. The Stacks pipeline had the highest reproducibility, with an error rate prior to filtering on reproducibility of 5.9%, which reduced to 2.9% after filtering out loci with poorest reproducibility. The error rate between technical repeats was 12.3% for both SAMtools and GATK. Error rates improved to 6.6 and 5.3% respectively after filtering on reproducibility.

### Between-population divergence

**Fig. 2:**
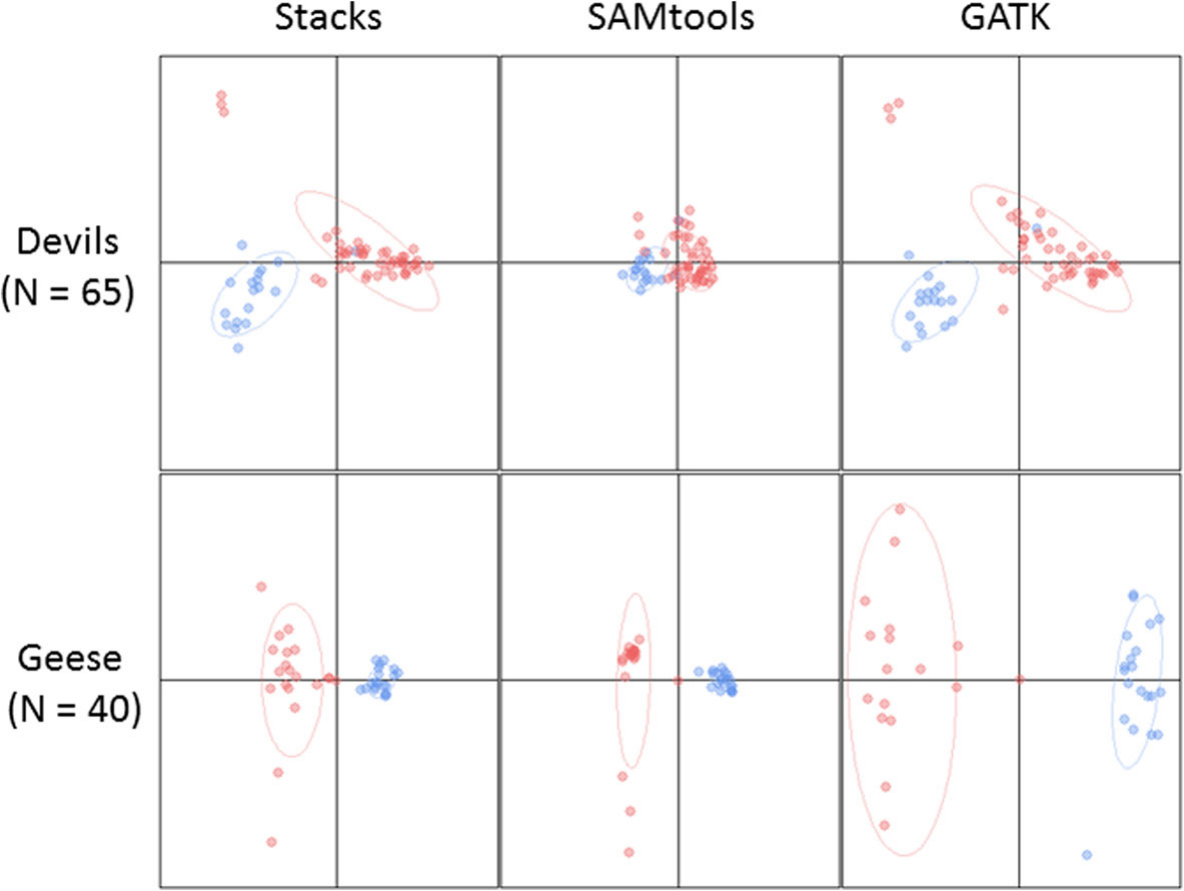
PCoAs of the two datasets after processing through three pipelines with a call rate of 70% and the custom R script as outlined in Fig. 1. For devils, red is the “west” (*N* = 47) and blue is the “east” (*N* = 18) population. For goose, red is the “Iceland” (*N* = 20) and blue is the “Denmark” (*N* = 20) population. Inertia ellipses illustrate groupings and do not necessarily indicate confidence

All three pipelines recovered the expected population structuring of both study species, with some variation among analysis methods. For both species, differentiation visualised using a principle coordinates analysis (PCoA) was clearest with the GATK pipeline, relative to the Stacks and SAMtools pipelines (Fig. 2). For devils, we also reanalysed our dataset with the addition of *N* = 66 captive animals (with a mixture of genetic heritage) and found that these fell intermediate to the two major populations, as expected (Additional file 1: Figure S3).

When analysed utilising pairwise F_ST_, we saw higher differentiation between our two major populations for devil than for goose (Table 3). Nevertheless, patterns across the three analysis methods were similar for both species: data processed by all three pipelines provided F_ST_ values that were similar (Table 3). These findings are consistent with our PCoA results, described above. Both species showed evidence of statistically significant population differentiation (Table 3).

**Table 3:** Population pairwise F_ST_ values for each analysis with 95% confidence intervals generated over 2000 bootstraps. In devils, Pop1 refers to the Western population (*N* = 47), Pop2 refers to the Eastern population (*N* = 18), and Pop3 refers to the insurance population (*N* = 66). In geese, Pop1 refers to the Iceland population (*N* = 20) and Pop2 refers to the Denmark population (*N* = 20)

Dataset	Pipeline	Pop 1:Pop 2	Pop 1:Pop 3	Pop 2:Pop 3
Devil (70% call rate)	Stacks	0.100 (0.090, 0.110)	0.030 (0.027, 0.034)	0.025 (0.021, 0.029)
	SAMtools	0.071 (0.056, 0.088)	0.019 (0.014, 0.025)	0.025 (0.017, 0.033)
	GATK	0.094 (0.084, 0.103)	0.029 (0.026, 0.033)	0.025 (0.021, 0.029)
Devil (30% call rate)	Stacks	0.091 (0.084, 0.100)	0.026 (0.023, 0.029)	0.025 (0.021, 0.029)
	SAMtools	0.067 (0.057, 0.078)	0.026 (0.022, 0.030)	0.015 (0.011, 0.021)
	GATK	0.091 (0.083, 0.099)	0.028 (0.025, 0.031)	0.026 (0.022, 0.030)
Goose (70% call rate)	Stacks	0.034 (0.032, 0.035)		
	SAMtools	0.038 (0.036, 0.039)		
	GATK	0.033 (0.032, 0.033)		
Goose (30% call rate)	Stacks	0.017 (0.016, 0.019)		
	SAMtools	0.092 (0.091, 0.093)		
	GATK	0.046 (0.045, 0.047)		

Each analysis method produced a varying amount of missing data (Table 1), but filtering less stringently (30% vs 70% call rate) to allow more missing data (and thus a greater number of loci) did not generally change the qualitative interpretation of our results by PCoA nor F_ST_ for either species. The exception is the Stacks analysis for goose, where the inclusion of many thousands more SNPs with low call rate obscured population inference (Additional file 1: Figure S4).

## Discussion

We examined population genetic inferences drawn from RRS data for two very different species with reference genomes using three analytical pipelines. Reference-aligned RRS analyses are poised to become much more common as a greater number of reference genomes become available. Genomes are no longer restricted to model species, and global initiatives such as the Earth Biogenome Project [31] aim to sequence all eukaryotic life, whilst targeted initiatives focus either on regionally important species (such as the Oz Mammals Genome Project [32]) or on particular taxa (such as the Birds 10K Project [33]). With an increasing proliferation of reference genomes, researchers skilled in the use of WGS alignment and assembly software (such as SAMtools [10] and GATK [11]) may prefer to use these tools when expanding their studies to include population-level RRS data. However, our results demonstrate the utility of purpose-built RRS pipelines with reasonable computational demands (such as combining Stacks with our custom R script) intended for use in non-model organisms.

Although all of the analytical pipelines we examined were able to detect genetic structure between the two populations of both species, there were differences in the resultant datasets. Due to the greater number of SNPs obtained, GATK may perform better for conducting analyses such as genome-wide associations that require a high marker density, however we note that computational resources required may be a limiting factor for use of GATK when studying non-model organisms (Table 1). Both SAMtools and GATK had higher initial error rates than Stacks, which could impact reliability for individual-level analyses, although our custom R script allows SNPs to be filtered out based on reproducibility to improve error rates, if replicates are performed. Stacks produced a comparable number of SNPs to GATK for devils, but far fewer for geese, and yet performed similarly well in detection of population structure in both species, with far less computational investment (Table 1, Fig. 2).

For both species, we observed a low percentage of shared loci across pipelines, which may introduce a source of ascertainment bias if extending a study to include more samples and using a prior set of defined loci. This observation may raise a potential red flag for many types of analyses (such as estimating allele frequencies or calculating linkage disequilibrium [34]), although it did not impact the population structure analyses we conducted here. Nevertheless, we note that genotype concordance across the shared loci was high. The tighter clustering of the two devil populations demonstrated by the SAMtools PCoA and the lower estimations of pairwise F_ST_ relative to Stacks and GATK, is likely influenced by the greater proportion of heterozygous genotype calls in that dataset. The apparent over-representation of heterozygous genotype calls in SAMtools can of course be addressed with additional data filtering which would be specific to each study so should be parameterised at the outset in future population genetic studies. Considering compute time and downstream population inferences, Stacks combined with the custom R script was the best performer of the three software packages we tested, and provided results that were independent of number of loci or percentage missing data for devils, but was influenced by missing data for geese.

In this study, we compared analysis pipelines using real datasets for two very different study species. Tasmanian devils are known to have low genetic diversity [27] and their numbers are declining due to DFTD [22]. The pink-footed goose, on the other hand, has higher genetic diversity and an expanding population [8]. As shown here, there are differences between the three pipelines observed in the PCoAs and pairwise F_ST_ comparisons. These may result in different recommendations, which may impact the genetic outcomes of the populations in question. We used the same parameters for each species for the purpose of comparison and note that our MAF thresholds may not be suitable for both populations given expected levels of diversity and sample sizes. The sample sizes were quite different and may have resulted in more alleles being sampled in the devil dataset, which has likely influenced population-level results [35]. We recommend MAF thresholds are parameterised at the outset of studies using RRS approaches.

Here we provide researchers with a customisable R pipeline (Additional file 2) that can be used for downstream analysis with data outputs in VCF format from any of these, or similar, software packages. The R pipeline works with VCF outputs from either initial alignment to a reference genome or de novo assembly and SNP calling. Our script allows for flexibility in choosing filtering thresholds by visual assessment of SNP data, as appropriate thresholds will differ between species, genotyping methods and downstream applications [36]. Filtering options include minimum read depth of both alleles (a feature that can be controlled in de novo alignment in Stacks with the -m parameter, but which is not implemented within Stacks for reference alignment), coverage difference, call rate, minor allele frequencies (MAF), heterozygosity and potentially sex-linked SNPs (based on XX/XY sex determination, though this could easily be reversed for ZZ/ZW organisms). An additional feature designed specifically to make use of the technical replicates performed by DArT PL is the reproducibility filter and error rate calculation, which can be extended to any RRS project where replicates have been used. The dartR package [37] contains functions for many of these filtering steps, however requires the proprietary DArT PL results spreadsheet as input for full functionality. Our custom R script can reproduce metrics provided by DArT PL from user-processed data, including SNP data from other RRS methods, allowing researchers to fully customise their analytical pipelines. The R script can be run on a standard personal computer in most scenarios, or on high performance computers, as is required with the thousands of SNPs output from GATK. We have specifically designed this pipeline so that researchers who work closely with conservation managers [38] can use genomic data to assist in making informed management decisions for species of conservation concern.

## Conclusion

While all pipelines performed well, they each have pros and cons which differ depending on the diversity present in the population and the amount of missing data. Stacks was less than optimal when missing data levels were high for goose as the populations could no longer be discriminated. SAMtools did not perform as well when the number of SNPs were low for devils so the diversity present was not great enough to discriminate between the populations as well as Stacks and GATK. GATK performed well but computational burden may exclude its use in some species of conservation concern where access to high performing compute resources may be limited and management decisions need to be made quickly following data collection. For our datasets, the Stacks pipeline combined with our custom R script is a robust and computationally efficient method for analysis of RRS data for both conservation-dependent and widespread species.

## Methods

### Datasets

Devil RRS data were obtained using DArTseq following [39], with full details provided at Additional file 1: Supplementary Methods (see Additional file 1: Figure S5 for sample quality). The restriction enzyme combination used was PstI-SphI, with fragments sequenced on an Illumina HiSeq 2500 as 77-bp single-end reads. Our devil dataset included animals originating from Western Tasmania (“Population 1”, *N* = 47) and Eastern Tasmania (“Population 2”, *N* = 18). In a further analysis we also considered data from *N* = 66 captive animals, which collectively comprised a mix of these two source populations and offspring thereof (“Population 3”). Methods for the goose RRS are reported at [8]. In brief, a ddRAD protocol was used with restriction enzymes Pst-HF and MSp1, and libraries sequenced on an Illumina HiSeq 2500 as 79-bp paired-end reads. We used data [40] for the Iceland (“Population 1”, *N* = 20) and Denmark (“Population 2”, N = 20) sites, as reported in [8].

#### Data cleaning

Stacks *process_radtags* was used on both devil and goose datasets to clean reads, removing those with any uncalled bases or low quality scores prior to aligning, and remove barcodes if necessary (devil data only, goose data already de-barcoded; see Additional file 1: Supplementary Methods).

### Alignment to reference genomes

#### Stacks pipeline

For both species, we used the Burrows-Wheeler aligner (BWA) v0.7.15 ‘aln’ function [41] to align single-end reads (devil) or paired-end reads (goose) following [14] to the respective reference genome [24, 29]. For our devil data, bias in per base sequence content was detected in the first 5 bases of reads (adaptor region) with FastQC so these were trimmed during the genome alignment step (−B 5) to remove the restriction enzyme cut site (PstI-HpaII). The BWA ‘samse’ function (devil, single-end reads) or ‘sampe’ function (goose, paired-end reads) was used to generate alignments in SAM format, which were converted to BAM format and ordered and indexed using SAMtools v1.6. Cleaned, trimmed, aligned data were then used as input for further analyses.

#### SAMtools and GATK pipelines

For our devil data, the first 5 bases were trimmed prior to alignment using bbDUK [42]. The ‘mem’ function in BWA was used to align reads following best practise guidelines [15], to the devil reference genome [24] followed by the SAMtools ‘sort’ function [10] to sort by genomic coordinate. Local realignment around indels was conducted using GATK IndelRealigner [11]. For our goose data, cleaned reads were aligned to the pink-footed goose genome [29] with BWA ‘mem’ followed by SAMtools sort and local realignment with GATK as per the devil data.

### Calling loci

Our three bioinformatic pipelines use slightly different methods to identify SNPs. To summarise, Stacks builds a catalogue of loci grouped across individuals [12], and applies a Bayesian maximum-likelihood approach developed by [43] that incorporates population genotype frequency information. GATK and SAMtools implement Bayesian approaches to call genotypes. GATK considers all reads covering a locus, as well as expected heterozygosity, to compute the posterior probability of a genotype [11]. SAMtools additionally includes Hidden Markov models to calibrate SNP calls using base alignment quality (BAQ) scores [44, 45].

#### Stacks pipeline

We used the Stacks v2.0b pipeline to process the sorted BAM files. The ‘gstacks’ module was run with default parameters (--model marukilow and --var-alpha 0.05) to create a catalogue of SNPs across our sample set as a single population. We ran the ‘populations’ module with the following parameters: a minimum call rate of 70% (−r 0.70), a maximum observed heterozygosity of 70% for devils (--max_obs_het 0.70) or 80% for goose (a higher threshold was chosen due to the much lower sample size), a minimum minor allele frequency (MAF) of 0.01 (--min_maf 0.01), and the --write_random_snp flag to randomly select only one SNP per locus.

#### SAMtools pipeline

SNPs were called from the realigned, sorted BAM files using SAMtools mpileup [10] with minimum base and mapping quality scores of 30. The coefficient for downgrading mapping quality of reads with excessive mismatches was set to 50 and bcftools call -m 6 was used to set a minimum depth of six reads to call a locus. This value was chosen to most closely simulate the Stacks parameter m = 3, which is minimum depth to call an allele, hence this was doubled to equate to minimum depth to call a locus. BCFtools merge [10] was used to merge single sample VCFs into a multi-sample VCF and filter on genotyping rate (min 70%, similar to Stacks -r) and MAF of 1% with VCFtools [46], to reflect the values used in the Stacks pipeline.

#### GATK pipeline

The realigned, sorted BAM files were used as input into GATK’s HaplotypeCaller [11] to produce individual gvcf files that were input into GATK’s GenotypeGVCFs to create a multi-sample gvcf file. VCFtools was again used to conduct preliminary data filtering using the same parameters as the SAMtools pipeline.

#### Custom R script

Within our custom R script (Additional file 2), we converted the VCF files from each of the three pipelines for the two species using the vcfR package [47] in order to extract the genotypes and associated metadata such as read depth. We further filtered the SNP set on average allelic depth, coverage difference, reproducibility and sex-linked SNPs. For SAMtools and GATK datasets, we also filtered on maximum observed heterozygosity as per the parameters used in Stacks. We set a minimum average read depth for both the reference and SNP allele as 2.5×. We calculated coverage difference as the percentage difference at each SNP between the read depth of the reference allele and SNP allele, and used a coverage difference of ≤80% as our cut-off. DArT PL performs technical replicates during the sequencing process, so for our devil dataset we calculated a measure of reproducibility as the genotype call error rate at each SNP between technical replicates once missing data is removed and filtered at > 85% reproducibility. We then recalculated error rate post-filtering. The goose dataset did not have replicates available for calculation of error rates or filtering on reproducibility.

In mammals, females are the homogametic sex with two X chromosomes, and males are heterogametic XY, whilst in birds females are heterogametic ZW and males are homogametic ZZ. We had accurate sex data for all devil samples and could therefore identify and filter out SNPs that may be sex-linked if no heterozygotes were present in the heterogametic sex but at least one heterozygote was present in the homogametic sex. We note however that this is a stringent filter and could be adjusted for sequencing errors. We did not have this information for goose and so did not apply any further filtering in this respect.

### Within-population diversity

The three resulting SNP datasets (Stacks, SAMtools and GATK) for each species were assessed for their ability to examine our study populations using a set of markers mapped to the genome. Data filtering and transformations were conducted using the custom R script for all datasets. For each of the datasets, summary statistics of observed (H_O_) and expected heterozygosity (H_E_) across loci were calculated using the adegenet package for R [48, 49]. The multilocus observed heterozygosity of individual devils (MLH) was calculated as a proportion of heterozygous loci across each individual. We extracted the shared loci between each pipeline for both species and used the ‘merge’ function in PLINK [50] to identify concordance rates between genotype calls across pipelines and output differing genotype calls for comparison.

### Between-population divergence

We performed principle coordinate analyses (PCoA) to discriminate population structuring and genetic clustering in the adegenet and ade4 [51] packages. This method calculates squared pairwise Euclidean distances between individuals allowing visualisation of population differentiation. PCoAs were run using population information to examine the structuring between “west”, “east”, or “IP” (insurance population, captive-born) samples for devil, and Iceland and Denmark for goose. For devils, two different analyses were performed for each of the three pipelines, the first including all individuals sequenced (*N* = 131), and the second only the founding wild-born individuals (*N* = 65). For devil samples with a technical replicate (*N* = 35), the sample with the least missing data from the SAMtools pipeline was selected (same sample selected across all pipelines). Pairwise fixation indices (F_ST_) were calculated using the StAMPP package for R [52], with 95% confidence intervals calculated via 2000 bootstraps across loci.

### Impact of missing data

For both species, we refiltered all three pipelines less stringently (genotyping rate of 30% rather than 70%) to examine the impacts of missing data on population inference. Calculation of summary statistics and F_ST_, and visualisation with PCoA were performed as above on the less stringently filtered SNP datasets.

## Additional files

Additional file 1: Supplementary Methods: Tasmanian devil reduced-representation sequencing. **Table S1.** Summary statistics for the resultant SNP loci datasets of three pipelines, filtered less stringently at a higher allowable missing data (30% call rate; *cf* Table 1), for Tasmanian devil (*N* = 131) and pink-footed goose (*N* = 40). **Figure S1.** Ratios of genotype calls between the three different pipelines for devils and geese. **Figure S2.** Venn diagram depicting number of shared loci between the three different pipelines for (a) devil and (b) goose. **Figure S3.** PCoA of the devil dataset only for the three pipelines, considering all three populations. Row one shows data processed with a call rate of 70%, row two shows data processed less stringently with a call rate of 30%. **Figure S4.** PCoAs of the two datasets after processing through three pipelines filtered less stringently, allowing more missing data (30% call rate). **Figure S5.** a) Gel image example of sample quality from 1 (highest) to 8 (no apparent DNA); b) - d) Gel quality rank (rank 7 and 8 not included as too low quality to include in study) vs. the amount of missing data of a sample for the b) Stacks, c) SAMtools and d) GATK pipelines. (ZIP 346 kb)

Additional file 2: Custom R script. (TXT 17 kb)

## Data Availability

Sequence data for devils has been deposited on the NCBI SRA (BioProject no. PRJNA540395; sample SRA accessions: SRR9001456-SRR9001623). Goose data is available on the NCBI SRA (BioProject Accession no. PRJNA400851/SRA accession SRP116633) and Dryad (10.5061/dryad.c4r81).

## Abbreviations

BAM: Binary alignment map
BAQ: Base alignment quality
BWA: Burrows-wheeler aligner
CPU: Central processing unit
ddRAD: Double digest restriction-site associated DNA
DFTD: Devil facial tumour disease
F_ST_: Fixation index
GATK: Genome analysis toolkit
GBS: Genotyping-by-sequencing
H_E_: Expected heterozygosity
H_O_: Observed heterozygosity
MAF: Minor allele frequency
MLH: Multilocus observed heterozygosity
NGS: Next-generation sequencing
PCoA: Principle coordinates analysis
RADseq: Restriction-site associated DNA sequencing
RRS: Reduced-representation sequencing
SAM: Sequence alignment map
SNP: Single nucleotide polymorphism
VCF: Variant call format
WGS: Whole genome sequencing
