# Supplementary material for: From reference genomes to population genomics: comparing three reference-aligned reduced-representation sequencing pipelines in two wildlife species
Source: BMC Genomics. 2019 Jun 3;20:453. doi: 10.1186/s12864-019-5806-y (PMC6547446; doi:10.1186/s12864-019-5806-y)
Supplement: Supplementary file 1 — Supplementary Methods: Tasmanian devil reduced-representation sequencing. Table S1. Summary statistics for the resultant SNP loci datasets of three pipelines, filtered less stringently at a higher allowable missing data (30% call rate; cf Table 1), for Tasmanian devil (N = 131) and pink-footed goose (N = 40). Figure S1. Ratios of genotype calls between the three different pipelines for devils and geese. Figure S2. Venn diagram depicting number of shared loci between the three different pipelines for (a) devil and (b) goose. Figure S3. PCoA of the devil dataset only for the three pipelines, considering all three populations. Row one shows data processed with a call rate of 70%, row two shows data processed less stringently with a call rate of 30%. Figure S4. PCoAs of the two datasets after processing through three pipelines filtered less stringently, allowing more missing data (30% call rate). Figure S5. a) Gel image example of sample quality from 1 (highest) to 8 (no apparent DNA); b) - d) Gel quality rank (rank 7 and 8 not included as too low quality to include in study) vs. the amount of missing data of a sample for the b) Stacks, c) SAMtools and d) GATK pipelines. (ZIP 346 kb) [file 12864_2019_5806_MOESM1_ESM.zip › Pipelines - supplementary final.docx]

**Supplementary Material to “From reference genomes to population genomics: comparing three reference-aligned reduced-representation sequencing pipelines in two wildlife species”**

Belinda Wright^1*^, Katherine A. Farquharson^1*^, Elspeth A. McLennan^1^, Katherine Belov^1^, Carolyn J. Hogg^1^, Catherine E. Grueber^1,2^^

*1. The University of Sydney, School of Life and Environmental Sciences, Faculty of Science, Sydney, Australia.*

*2. San Diego Zoo Global, San Diego, USA*

** contributed equally*

*^ corresponding author*

belinda.wright@sydney.edu.au ; katherine.farquharson@sydney.edu.au ; elspeth.mclennan@sydney.edu.au ; kathy.belov@sydney.edu.au ; carolyn.hogg@sydney.edu.au ; catherine.grueber@sydney.edu.au

**Supplementary Methods: Tasmanian devil reduced-representation sequencing**

In response to concerns about the persistence of the devil in the wild, a captive insurance population was established in 2006 with the intake of 122 founders from across Tasmania [1]. Due to the progression of the disease, founding devils were only obtained from limited locations on the east coast or from the north-western region of Tasmania, causing distinct population structuring among the founding individuals [1]. To overcome concerns of potential inbreeding, founders were regularly paired with individuals from the opposing provenance resulting in mixed lineages among the insurance population [1]. The insurance population now consists of over 700 devils across 37 zoo-based facilities and free-range enclosures, one island (Maria Island) and a fenced peninsula (Forestier Peninsula) [2]. For this study, we selected 131 Tasmanian devil samples from our genomic DNA database, including 65 wild-caught individuals of both eastern and western origins (Fig 1), and 66 captive-born individuals from the Tasmanian devil insurance population with mixed lineages [1]. The wild-caught individuals included here were a subset of those used to establish the insurance population (i.e. population “founders”), for which sufficient archival DNA was available for analysis.

Ear biopsies collected in 70% ethanol, or 1 mL whole blood in EDTA, have been collected from Tasmanian devils by the Save the Tasmanian Devil Program, or participating zoos, for management purposes since the commencement of the insurance population. These archival samples varied in quality, assessed visually via gel electrophoresis (see below). DNA was extracted using either a modified phenol/chloroform protocol [3] or commercial DNA extraction kit (Qiagen DNeasy Blood & Tissue Kit). DNA sample quality was assessed using a NanoDrop to measure DNA concentration, and by visualisation via agarose gel (0.8%, 90V for 30 minutes) to measure concentration and fragmentation. Extractions were scored from 1 - 8, with a strong, clear band on the gel given a ranking of 1 - 2 ‘high quality’, 3 - 4 is a moderate-strength band, 5 - 6 is a weak fragmented ‘poor quality’ band and 7 - 8 is no evidence of a band or DNA ‘very poor quality’ (see Supplementary Figure S5a for examples). Of our 131 unique samples, 26 (19.8%) were rated ‘high’ quality, 49 (28.6%) ‘moderate’ quality, and 56 (32.6%) ‘poor’ quality. None were rated ‘very poor’. There was no clear trend in sample quality vs. sequencing quality, measured as proportion of missing data/total SNPs (Supplementary Figure S5b-d) across the three pipelines.

Of the many RRS techniques available, DArTseq™ (Diversity Arrays Technology Pty Ltd, hereafter DArT PL) is particularly well-used in Australia for varied applications including management of selective breeding programs, genetic mapping, and population genetics studies [4-6]. Initially developed for use in commercially important crop species, the approach has since been applied to diverse wildlife including mammals [7], reptiles [8], amphibians [5] and fish [9-11]. The restriction enzyme combination used by DArT PL for our dataset was PstI-SphI, with fragments sequenced on a HiSeq 2500 as 77-bp single-end reads. DArT PL also performed technical sample replicates, resulting in raw sequences from 166 samples for analysis. Following sequencing, DArT PL returns results from their proprietary data filtering pipeline, DArTSoft14, as a Microsoft Excel spreadsheet. Recently, the dartR package [12] in R [13] has been developed for filtering and analysis of the DArT PL spreadsheets. In this study however, we processed the raw sequencing reads also provided by DArT PL.

Raw data were processed using the ‘process_radtags’ module of Stacks v2.0b [14] with the flags --disable_rad_check and --inline_null (as a single inline barcode was used) to remove barcodes (4bp - 8bp) for each sequencing lane, check for adapter contamination and clean data of reads containing uncalled bases. We performed checks of the log files to ensure all samples had a reasonable number of reads. Cleaned reads were then investigated using FastQC [15] to visually check for sequencing errors and to determine if reads needed to be trimmed. These cleaned reads were used as input for further processing and analysis in all three pipelines.

**Table S1** Summary statistics for the resultant SNP loci datasets of three pipelines, filtered less stringently at a higher allowable missing data (30% call rate; *cf* Table 1), for Tasmanian devil (N = 131) and pink-footed goose (N = 40), including the total number of loci (total loci), the average number of loci sequenced across individuals (mean loci), the amount of missing data (%), the calculated error rates (%), the mean observed heterozygosity across loci (H_O_), the mean expected heterozygosity across loci (H_E_), and the average multilocus heterozygosity of individuals (MLH)

| Dataset | Pipeline | Total loci | Mean loci (min; max) | % missing | Error rate^1^  (%) | H_O_  (± SD) | H_E_  (± SD) | MLH  (± SD) |
| --- | --- | --- | --- | --- | --- | --- | --- | --- |
| Devils | Stacks | 2,537 | 1,773.4 (680; 2,186) | 30.1 | 1.9 | 0.215 (0.168) | 0.260 (0.167) | 0.208 (0.042) |
|  | SAMtools | 786 | 479.4 (172; 569) | 39.0 | 5.8 | 0.342 (0.186) | 0.357 (0.121) | 0.328 (0.090) |
|  | GATK | 2,450 | 1834.8 (682; 2192) | 25.1 | 4.9 | 0.163 (0.147) | 0.251 (0.163) | 0.167 (0.033) |
| Geese | Stacks | 139,979 | 86,844.2 (1616; 128,458) | 38.0 | NA | 0.163 (0.157) | 0.207 (0.153) | 0.134 (0.036) |
|  | SAMtools | 146,599 | 79,440.6 (3407; 109,403) | 45.8 | NA | 0.290 (0.181) | 0.351 (0.134) | 0.268 (0.117) |
|  | GATK | 601,707 | 412,978.5 (10,584; 553,941) | 31.4 | NA | 0.137 (0.125) | 0.216 (0.155) | 0.126 (0.041) |

^1^ Error rates could not be calculated for the pink-footed goose dataset as no replicates were included in the current analysis. Error rate is calculated after filtering on SNPs with > 85% reproducibility, so is lower than initial error rates.

**Captions to Supplementary Figures**

**Figure S1** Ratios of genotype calls between the three different pipelines for devils and geese. Blue indicates the most frequent homozygotes, orange indicates heterozygotes and grey the least frequent homozygotes. Note that SAMtools and GATK are able to report those homozygotes that match the reference or alternate allele but Stacks assigns the most frequent allele as the reference allele, hence how these genotypes are referred to here.

**Figure S2** Venn diagram depicting number of shared loci between the three different pipelines for (a) devil and (b) goose.

**Figure S3** PCoA of the devil dataset only for the three pipelines, considering all three populations. “West” (red) and “east” (blue) are the wild-born founding individuals (N = 65). “IP” (green) are the captive-born insurance population individuals. Row one shows data processed with a call rate of 70%, row two shows data processed less stringently with a call rate of 30%. Inertia ellipses illustrate groupings and do not necessarily indicate confidence.

**Figure S4** PCoAs of the two datasets after processing through three pipelines filtered less stringently, allowing more missing data (30% call rate). For devils, red is the “west” and blue is the “east” population. For goose, red is the “Iceland” and blue is the “Denmark” population. Inertia ellipses illustrate groupings and do not necessarily indicate confidence.

**Figure S5** a) Gel image example of sample quality from 1 (highest) to 8 (no apparent DNA); b) - d) Gel quality rank (rank 7 and 8 not included as too low quality to include in study) vs. the amount of missing data of a sample for the b) Stacks, c) SAMtools and d) GATK pipelines. Boxplots are scaled with the width proportional to the number of samples within the gel quality rank (range 9 to 44).

**Literature cited in Supplementary Material**

1. Hogg CJ, Ivy JA, Srb C, Hockley J, Lees C, Hibbard C *et al*: Influence of genetic provenance and birth origin on productivity of the Tasmanian devil insurance population. Conserv Genet 2015; 16(6):1465-1473.

2. Hogg CJ, Lee AV, Srb C, Hibbard C: Metapopulation management of an Endangered species with limited genetic diversity in the presence of disease: the Tasmanian devil *Sarcophilus harrisii*. Int Zoo Yearb 2017; 51(1):137-153.

3. Sambrook J, Russell DW: Purification of nucleic acids by extraction with phenol: chloroform. CSH Protoc 2006; 2006(1):pdb. prot4455.

4. Ren R, Ray R, Li P, Xu J, Zhang M, Liu G *et al*: Construction of a high-density DArTseq SNP-based genetic map and identification of genomic regions with segregation distortion in a genetic population derived from a cross between feral and cultivated-type watermelon. Mol Genet Genomics 2015; 290(4):1457-1470.

5. Lambert MR, Skelly DK, Ezaz T: Sex-linked markers in the North American green frog (*Rana clamitans*) developed using DArTseq provide early insight into sex chromosome evolution. BMC Genomics 2016; 17(1):844.

6. Baloch FS, Alsaleh A, Shahid MQ, Çiftçi V, de Miera LES, Aasim M *et al*: A whole genome DArTseq and SNP analysis for genetic diversity assessment in durum wheat from central fertile crescent. PloS One 2017; 12(1):e0167821.

7. Schultz AJ, Cristescu RH, Littleford-Colquhoun BL, Jaccoud D, Frère CH: Fresh is best: Accurate SNP genotyping from koala scats. Ecol Evol 2018; 8(6):3139-3151.

8. Melville J, Haines ML, Boysen K, Hodkinson L, Kilian A, Smith Date KL *et al*: Identifying hybridization and admixture using SNPs: application of the DArTseq platform in phylogeographic research on vertebrates. Royal Soc Open Sci 2017; 4(7):161061.

9. Donnellan SC, Foster R, Jung C, Huveneers C, Rogers P, Killian A *et al*: Fiddling with the proof: the Magpie Fiddler Ray is a colour pattern variant of the common Southern Fiddler Ray (*Rhinobatidae: Trygonorrhina*). Zootaxa 2015; 3981(3):18.

10. DiBattista JD, Travers MJ, Moore GI, Evans RD, Newman SJ, Feng M *et al*: Seascape genomics reveals fine-scale patterns of dispersal for a reef fish along the ecologically divergent coast of Northwestern Australia. Mol Ecol 2017; 26(22):6206-6223.

11. Pazmiño DA, Maes GE, Simpfendorfer CA, Salinas-de-León P, van Herwerden L: Genome-wide SNPs reveal low effective population size within confined management units of the highly vagile Galapagos shark (*Carcharhinus galapagensis*). Conserv Genet 2017; 18(5):1151-1163.

12. Gruber B, Unmack PJ, Berry OF, Georges A: dartr: An r package to facilitate analysis of SNP data generated from reduced representation genome sequencing. Mol Ecol Resour 2018; 18(3):691-699.

13. R Core Team: R: A language and environment for statistical computing. In., <https://www.R-project.org/>. Vienna, Austria: R Foundation for Statistical Computing; 2018.

14. Catchen J, Hohenlohe PA, Bassham S, Amores A, Cresko WA: Stacks: an analysis tool set for population genomics. Mol Ecol 2013; 22(11):3124-3140.

15. Andrews S: FastQC: a quality control tool for high throughput sequence data. In., <http://www.bioinformatics.babraham.ac.uk/projects/fastqc>; 2010.
