# Supplementary figures and images for: From reference genomes to population genomics: comparing three reference-aligned reduced-representation sequencing pipelines in two wildlife species
Source: BMC Genomics. 2019 Jun 3;20:453. doi: 10.1186/s12864-019-5806-y (PMC6547446; doi:10.1186/s12864-019-5806-y)

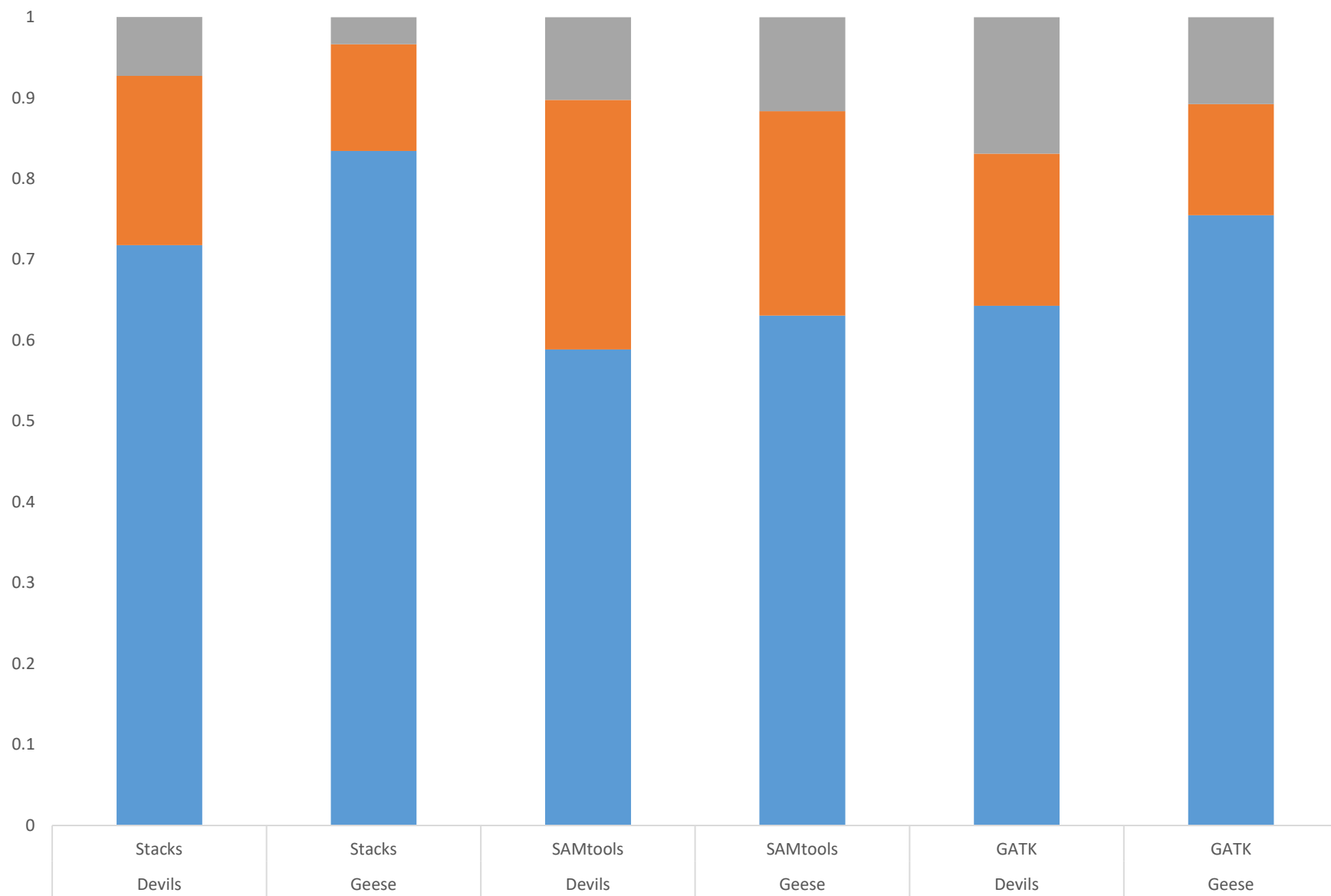

Supplement: Supplementary file 1 — Supplementary Methods: Tasmanian devil reduced-representation sequencing. Table S1. Summary statistics for the resultant SNP loci datasets of three pipelines, filtered less stringently at a higher allowable missing data (30% call rate; cf Table 1), for Tasmanian devil (N = 131) and pink-footed goose (N = 40). Figure S1. Ratios of genotype calls between the three different pipelines for devils and geese. Figure S2. Venn diagram depicting number of shared loci between the three different pipelines for (a) devil and (b) goose. Figure S3. PCoA of the devil dataset only for the three pipelines, considering all three populations. Row one shows data processed with a call rate of 70%, row two shows data processed less stringently with a call rate of 30%. Figure S4. PCoAs of the two datasets after processing through three pipelines filtered less stringently, allowing more missing data (30% call rate). Figure S5. a) Gel image example of sample quality from 1 (highest) to 8 (no apparent DNA); b) - d) Gel quality rank (rank 7 and 8 not included as too low quality to include in study) vs. the amount of missing data of a sample for the b) Stacks, c) SAMtools and d) GATK pipelines. (ZIP 346 kb) [file 12864_2019_5806_MOESM1_ESM.zip › Figure S1.pdf]

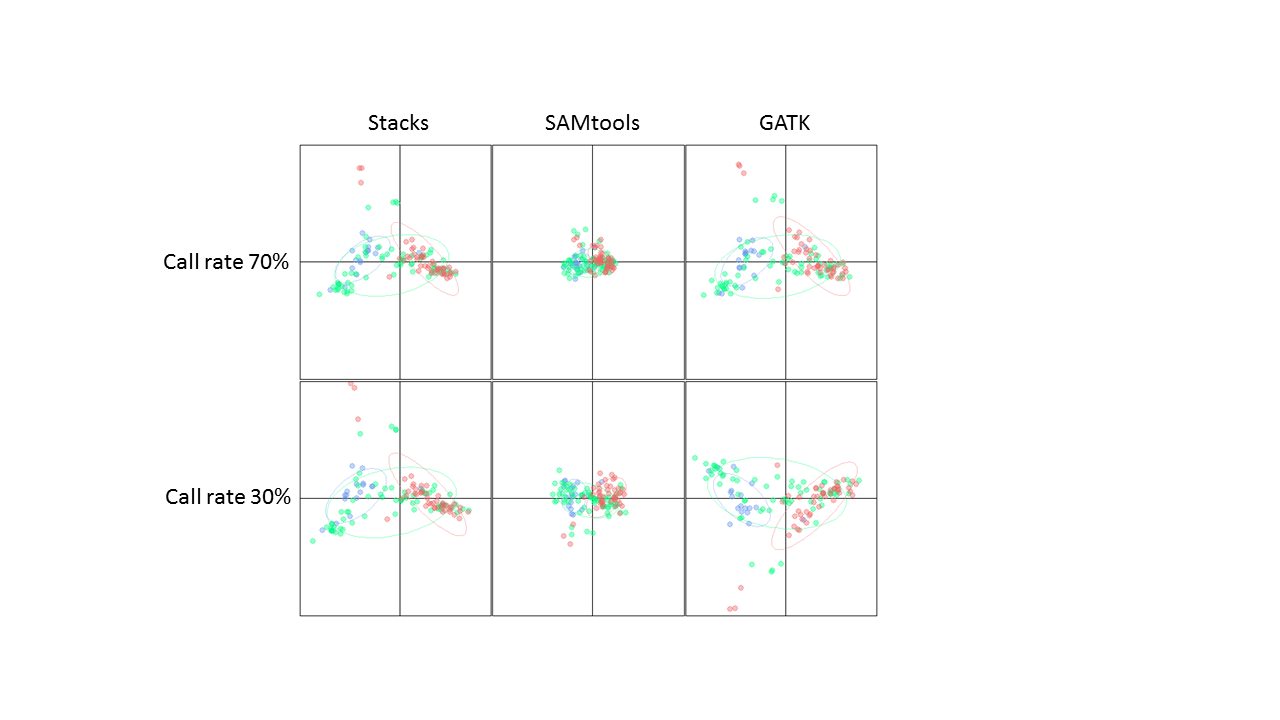

Supplement: Supplementary file 1 — Supplementary Methods: Tasmanian devil reduced-representation sequencing. Table S1. Summary statistics for the resultant SNP loci datasets of three pipelines, filtered less stringently at a higher allowable missing data (30% call rate; cf Table 1), for Tasmanian devil (N = 131) and pink-footed goose (N = 40). Figure S1. Ratios of genotype calls between the three different pipelines for devils and geese. Figure S2. Venn diagram depicting number of shared loci between the three different pipelines for (a) devil and (b) goose. Figure S3. PCoA of the devil dataset only for the three pipelines, considering all three populations. Row one shows data processed with a call rate of 70%, row two shows data processed less stringently with a call rate of 30%. Figure S4. PCoAs of the two datasets after processing through three pipelines filtered less stringently, allowing more missing data (30% call rate). Figure S5. a) Gel image example of sample quality from 1 (highest) to 8 (no apparent DNA); b) - d) Gel quality rank (rank 7 and 8 not included as too low quality to include in study) vs. the amount of missing data of a sample for the b) Stacks, c) SAMtools and d) GATK pipelines. (ZIP 346 kb) [file 12864_2019_5806_MOESM1_ESM.zip › figure S3.tif]

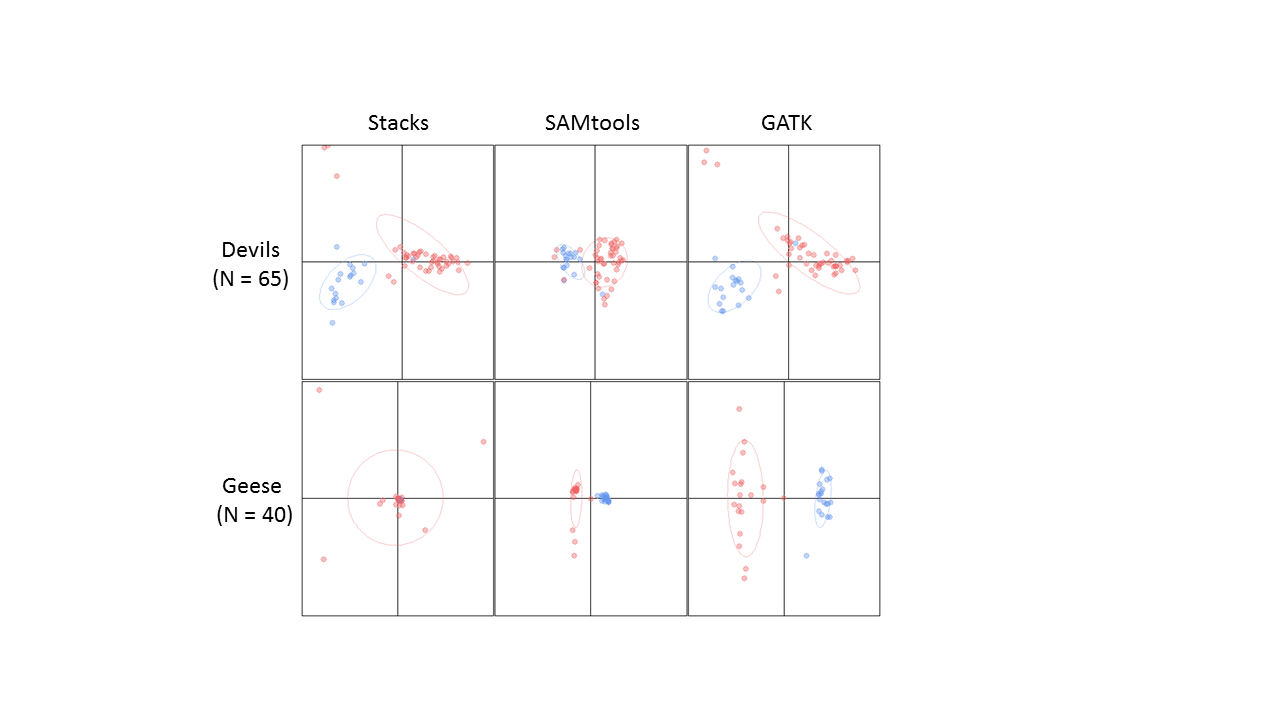

Supplement: Supplementary file 1 — Supplementary Methods: Tasmanian devil reduced-representation sequencing. Table S1. Summary statistics for the resultant SNP loci datasets of three pipelines, filtered less stringently at a higher allowable missing data (30% call rate; cf Table 1), for Tasmanian devil (N = 131) and pink-footed goose (N = 40). Figure S1. Ratios of genotype calls between the three different pipelines for devils and geese. Figure S2. Venn diagram depicting number of shared loci between the three different pipelines for (a) devil and (b) goose. Figure S3. PCoA of the devil dataset only for the three pipelines, considering all three populations. Row one shows data processed with a call rate of 70%, row two shows data processed less stringently with a call rate of 30%. Figure S4. PCoAs of the two datasets after processing through three pipelines filtered less stringently, allowing more missing data (30% call rate). Figure S5. a) Gel image example of sample quality from 1 (highest) to 8 (no apparent DNA); b) - d) Gel quality rank (rank 7 and 8 not included as too low quality to include in study) vs. the amount of missing data of a sample for the b) Stacks, c) SAMtools and d) GATK pipelines. (ZIP 346 kb) [file 12864_2019_5806_MOESM1_ESM.zip › figure S4.tif]

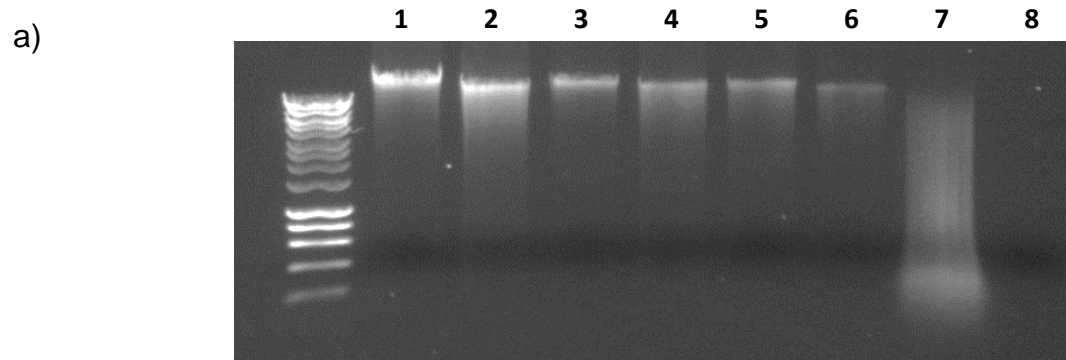

b) Stacks

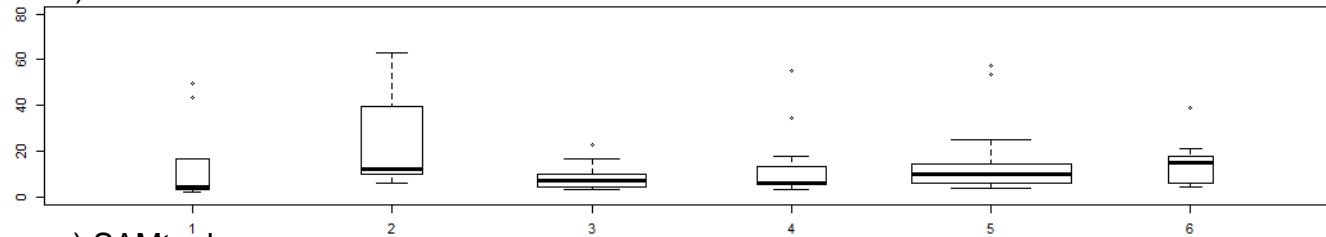

c) SAMtools

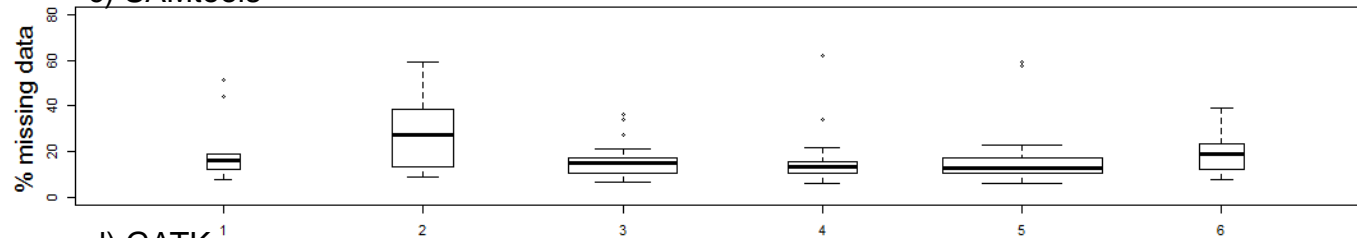

d) GATK

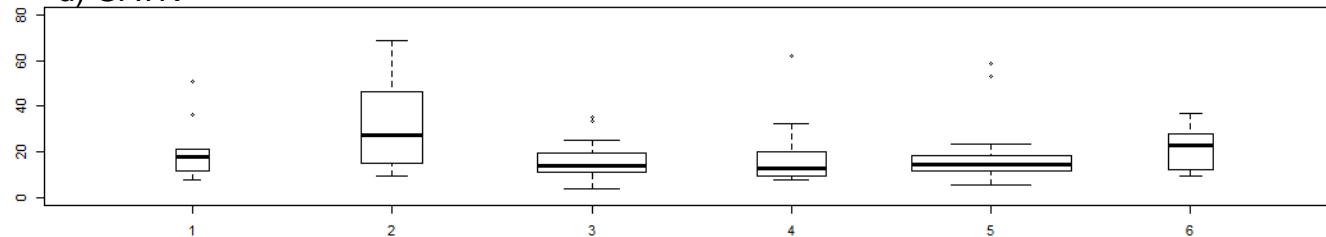

Gel quality rank

Supplement: Supplementary file 1 — Supplementary Methods: Tasmanian devil reduced-representation sequencing. Table S1. Summary statistics for the resultant SNP loci datasets of three pipelines, filtered less stringently at a higher allowable missing data (30% call rate; cf Table 1), for Tasmanian devil (N = 131) and pink-footed goose (N = 40). Figure S1. Ratios of genotype calls between the three different pipelines for devils and geese. Figure S2. Venn diagram depicting number of shared loci between the three different pipelines for (a) devil and (b) goose. Figure S3. PCoA of the devil dataset only for the three pipelines, considering all three populations. Row one shows data processed with a call rate of 70%, row two shows data processed less stringently with a call rate of 30%. Figure S4. PCoAs of the two datasets after processing through three pipelines filtered less stringently, allowing more missing data (30% call rate). Figure S5. a) Gel image example of sample quality from 1 (highest) to 8 (no apparent DNA); b) - d) Gel quality rank (rank 7 and 8 not included as too low quality to include in study) vs. the amount of missing data of a sample for the b) Stacks, c) SAMtools and d) GATK pipelines. (ZIP 346 kb) [file 12864_2019_5806_MOESM1_ESM.zip › Figure S5.pdf]
